# Controlling the shape of LiCoPO_4_ nanocrystals by supercritical fluid process for enhanced energy storage properties

**DOI:** 10.1038/srep03975

**Published:** 2014-02-05

**Authors:** Quang Duc Truong, Murukanahally Kempaiah Devaraju, Yoshiyuki Ganbe, Takaaki Tomai, Itaru Honma

**Affiliations:** 1 Institute of Multidisciplinary Research for Advanced Materials, Tohoku University, Sendai 980-8577, Japan

## Abstract

Lithium-ion batteries offer promising opportunities for novel energy storage systems and future application in hybrid electric vehicles or electric vehicles. Cathode materials with high energy density are required for practical application. Herein, high-voltage LiCoPO_4_ cathode materials with different shapes and well-developed facets such as nanorods and nanoplates with exposed {010} facets have been synthesized by a one-pot supercritical fluid (SCF) processing. The effect of different amines and their roles on the morphology-control has been investigated in detail. It was found that amine having long alkyl chain such as hexamethylenediamine played important roles to manipulate the shape of the nanocrystals by selective adsorption on the specific {010} facets. More importantly, the nanorods and nanoplates showed better electrochemical performance than that of nanoparticles which was attributed to their unique crystallographic orientation with short Li ion diffusion path. The present study emphasizes the importance of crystallographic orientation in improving the electrochemical performance of the high voltage LiCoPO_4_ cathode materials for Li-ion batteries.

Lithium-ion batteries offer promising opportunities for novel energy storage systems and future application in hybrid electric vehicles (HEV) or electric vehicles (EV) due to its low cost, stability, lightweight, low maintenance, high energy density and high power density^1^,^2^. Among a variety of cathode materials, lithium transition metal phosphates (LiMPO_4_, M = Mn, Fe, Co, Ni) with olivine structure have attracted extraordinary attention owing to the strong P-O covalent bond and the resulting thermodynamical and dynamical stability at high temperature and charge state. Particularly, LiFePO_4_ has been widely studied and commercially produced due to its eco-friendliness and thermal stability^3^,^4^. LiMnPO_4_ is another promising cathode material with a higher operating voltage at 4.1 V versus Li/Li^+^^5^,^6^. However, LiFePO_4_ is limited from its low discharge potential (3.4 V vs. Li/Li^+^) and LiMnPO_4_ suffers from Jahn-Teller distortion and large volume change during the charge-discharge cycle^5^,^6^. For these reasons, the lithium cobalt phosphate (LiCoPO_4_) has attracted attention since it offers both flat high potential (at approximately 4.8 V versus Li/Li^+^), good theoretical capacity (167 mAhg^−1^) and smaller structure volume change^7^,^8^. However, the major drawback with the LiCoPO_4_ cathodes is the poor lithium ion and electronic conductivity. Therefore, efforts have been made in recent years on improvement of the electrochemical performance of the LiCoPO_4_ by cationic doping, decreasing the particle size through various synthesis methods, and coating with electronically conducting agents^9^,^10^,^11^,^12^,^13^,^14^,^15^,^16^,^17^. Enhanced cyclic performance has been observed by partial substitution of Co or Li in LiCoPO_4_ structure by Fe, V or other divalent cation^18^,^19^,^20^. Down-sizing LiCoPO_4_ particles has been shown to improve the electrochemical performance due to a shortening of Li ion diffusion distance^16^,^17^. Therefore, nanorods and nanoplates are promising owing to the advantages such as fast charge and mass transport as well as enhancing the contact with the electrolyte and the resulting reaction kinetics. Furthermore, the crystallographic orientation has significant effect on the electrochemical properties. In the orthorhombic olivine structure, Li^+^ diffusion energy is orientation-dependent which is lowest for the pathway along the [010] channel^21^. It has been also observed the movement of Li ions in the *b* direction at the phase boundary by electron microscopy^22^, or by combining high-temperature powder neutron diffraction and the maximum entropy method^23^. Thus, it is accepted that lithium ions one-dimensionally diffuse along the *b* axis during the lithiation/delithiation reactions. The growth of nanorods along *c*-axis or nanoplates with thinnest part along [010] direction is highly desirable to obtain nanocrystals with high energy density and high power density.

Solution-based methods are appealing to control particles size, shape and morphology of nanocrystals. Attempts to use solution methods for controlled synthesis of LiCoPO_4_ have received a limited success^24^,^25^,^26^,^27^,^28^,^29^ and the effect of crystallographic orientation and particle morphology on the electrochemical performance has not been reported. In our recent works, we have developed novel supercritical fluid processes for controlled synthesis of various polyanion compounds^30^,^31^,^32^,^33^,^34^,^35^. The supercritical fluids have been considered to be unique reaction media for nanocrystal synthesis due to its adjustable physicochemical properties (density, viscosity, dielectric constant) as a function of temperature and pressure. The other advantageous properties including low interfacial tension, excellent wetting of surfaces, and high diffusion coefficients, makes supercritical fluid a potentially superior media for controlled synthesis of olivine lithium metal phosphates^34^,^35^,^36^. In this paper, we report a fully study on the crystal growth, characterization and investigation on crystallographic orientation and exposed facets of LiCoPO_4_ nanocrystals. We presented three examples of LiCoPO_4_ nanocrystals including nanoparticles, nanorods, nanoplates and investigated the effect of crystal shapes on the electrochemical performance of the cathode material. A maximum initial discharge capacity of 130 mAhg^−1^ at C/10 rate (77.8% theoretical capacity) was obtained for nanorod particles, while nanoplates showed higher rate capacity.

## Results

### Characterization of LiCoPO_4_ nanocrystals

The supercritical fluid conditions are beneficial for the control of hydrolysis and nucleation. Lee and Teja found that high nucleation rate and homogeneous crystallization in smaller size particles with narrow size distribution would proceeded due to the increased hydrolysis rate at high temperatures and reduced solubility due to the change in dielectric constant^36^. At the same time, organic molecules and nuclei can be homogeneously miscible at supercritical fluid conditions, leading facile surface functionalization of the synthesized nanocrystals.

**Figure 1:**
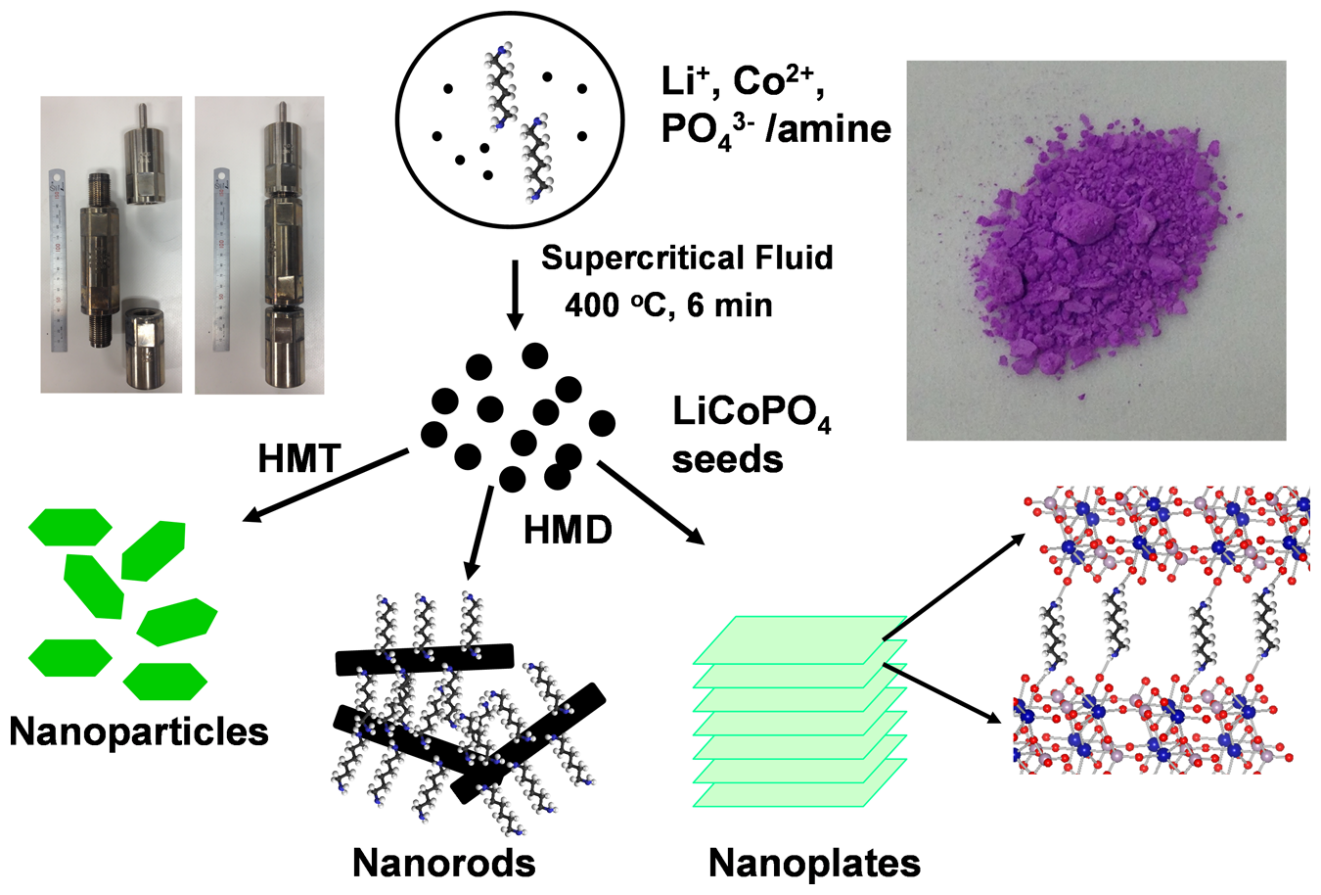
Schematic illustrations. The crystal growth and effect of amines on the morphology of the synthesized LiCoPO_4_ particles (HMT: hexamethylenetretramine; HMD: hexamethylenediamine). The insets shows the photograph images of supercritical fluid reactors (left) and LiCoPO_4_ powder obtained by supercritical fluid process (right).

The formation of the LiCoPO_4_ nanocrystals in supercritical ethanol can be explained as a three-step mechanism as schematically illustrated in Fig. 1. In the initial stage, rapid heating under the supercritical fluid promotes the supersaturation of reactant species, leading to the formation of LiCoPO_4_ seeds. In the second step, nuclei growth proceeds to produce small nanocrystals, which were further grown and assembled into different shaped crystals due to the effect of ambient solution. Under supercritical fluid condition, the particle size, shape and crystal orientation vary significantly with the change in reaction temperature, reaction time, solvent composition and additive concentration^30^,^31^,^32^,^33^,^34^,^35^. We therefore maintained reaction temperature, reaction time, solvent composition constant and tailored the additive and amount of additive to clarify the effect of additive on morphologies and effect of particle shape on the electrochemical performance. It was reported that LiCoPO_4_ could be formed under basic condition^24^,^25^,^26^,^27^,^28^,^29^. Furthermore, under high temperature and pressure condition, the growth rate of LiCoPO_4_ was very fast resulting in micron-size particles. Therefore, we used amines such as hexamethylenetetramine (HMT) and hexamthylenediamine (HMD) as a *in situ* OH^−^ sources to control the basic condition of the reaction solution as well as the growth rate of LiCoPO_4_ nanocrystals.

The X-ray diffraction (XRD) patterns of the synthesized LiCoPO_4_ particles by SCF are shown in Fig. S1. All diffraction peaks were indexed to orthorhombic *Pnma* space group with *a* = 10.2045 Å, *b* = 5.9213 Å, *c* = 4.7002 Å, in agreement with reported values^37^,^38^. It is evident from the XRD patterns that single phase of LiCoPO_4_ with olivine structure has been prepared.

**Figure 2:**
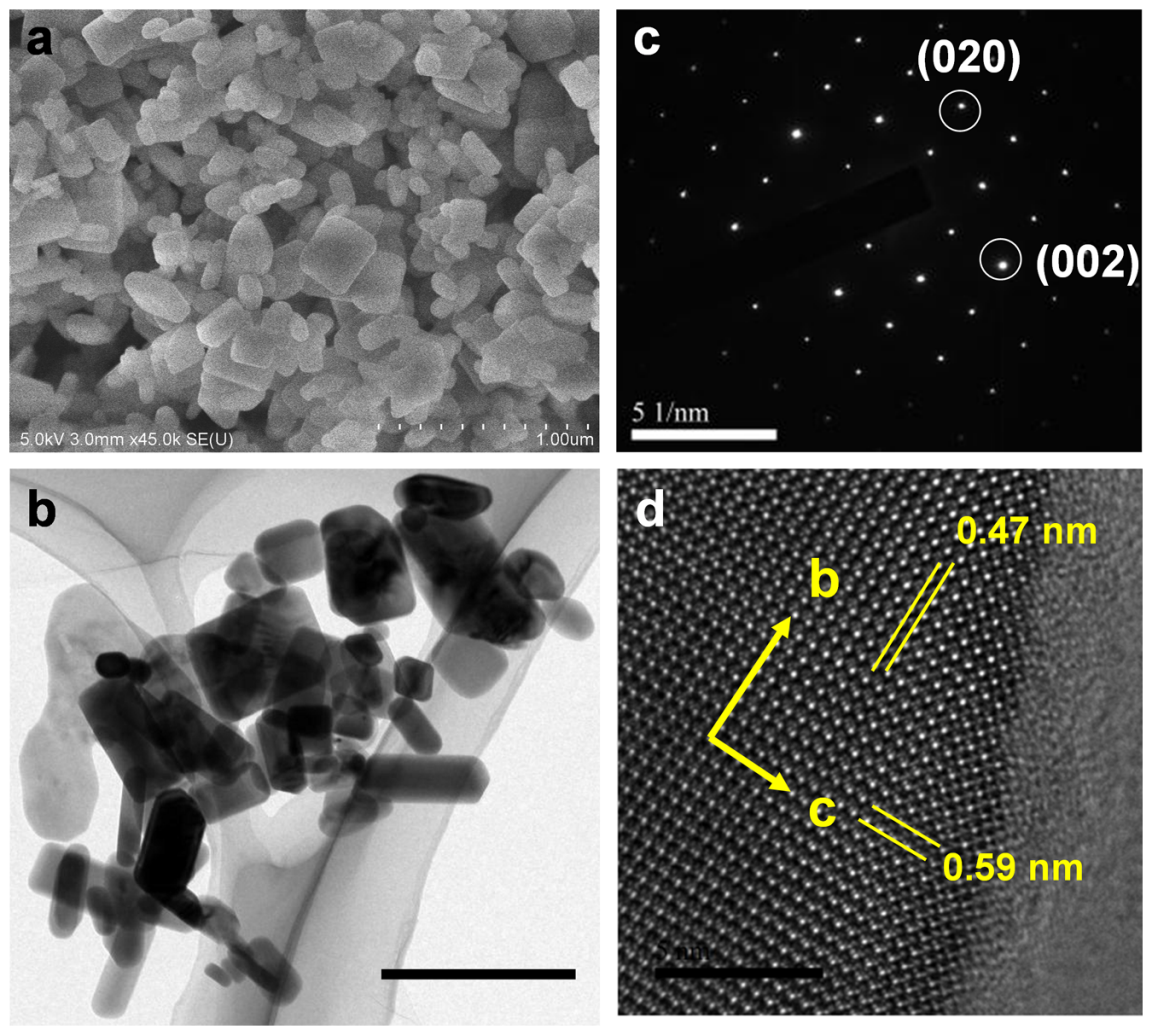
Nanoparticles synthesized with hexamethylenetretramine. (a) SEM image (scale bar = 1 μm); (b) TEM image (scale bar = 500 nm); (c) SAED pattern and (d) HRTEM image (scale bar = 5 nm).

The particle morphology was observed using scanning/transmission electron microscopes (S/TEM). Fig. 2a and b show SEM and TEM images of LiCoPO_4_ nanoparticles synthesized with 4 mmol HMT. The LiCoPO_4_ nanoparticles and submicron-sized particles with different shapes show diameters ranging from 100 nm to 300 nm evidenced from SEM image. Fig. 2c and d show the selected-area electron diffraction (SAED) pattern and HR-TEM image of a typical particle, respectively. SAED pattern taken from the particles with well-defined diffraction spots can be indexed to the [100] zone axis of single-crystal olivine structure, indicating that the particle is single crystalline in nature. The SAED pattern confirms that the crystal growth orientation is along the *bc* planes with exposed large {100} facets in the particle. The HR-TEM image in Fig. 2d clearly shows the interplanar spacing of (010) and (001) atomic planes. The lattice fringes spacing is 5.9 Å and 4.7 Å along [010] and [001] directions which is consistent with the unit cell parameter along the crystallographic *b, c* directions, *b* = 5.9213 Å and *c* = 4.7002 Å. A corresponding model of atomic arrangement of LiCoPO_4_ olivine structure projected along [100] direction is depicted in Fig. S2 which is in agreement with the observation shown in Fig. 2c, d. More importantly, the lattice fringes are aligned without any dislocation and the diffraction spots are uniform, indicating the high crystallinity of the obtained structures.

Furthermore, to verify the chemical composition of the synthesized particles, the energy dispersive X-ray spectroscopy (EDS) analysis and elemental mapping of the LiCoPO_4_ sample by STEM were conducted. EDS elemental mappings by STEM (Fig. S3) shows a uniform distribution of Co, P, O, and the EDS spectrum of the sample exhibits the characteristic peaks of Co, P, O, consistent with the LiCoPO_4_ phase purity determined by XRD and electron microscopy. No other element was detected in EDS analysis by STEM. These characterizations confirm the purity, homogeneity and uniform elemental distribution in the obtained particles.

It should be noted that when smaller amount of HMT (2 mmol) was used, the obtained particles is composed of inhomogeneous mixture of different sizes and shapes (Fig. S4a, b). When 10 mmol of HMT was added to the reaction solution, submicron-sized particles are formed (Fig. S4c, d). If the more amount of HMT was added to the solution, the mixture of blue and pink colored products was obtained, suggesting the formation of impurity phases. The uniform nanoparticles could be obtained with a suitable amount of HMT (4 mmol). Thus, HMT played an important role in controlling the size of the synthesized LiCoPO_4_ particles. It was reported that under elevated temperature, HMT was hydrolyzed to form ammonia and formaldehyde (Eq. 1, 2)^39^,^40^. The generated NH_3_, thus, provided the basic condition for the crystallization of LiCoPO_4_. Furthermore, the *in situ* release of ammonia and resulting OH^−^ anions in the solution restrained the crystal growth rate, resulting in the formation of nanoparticles. To ensure the importance of *in situ* OH^−^ generation, the control experiment using different amounts of NH_3_ were carried out. Micron-sized particles were obtained, as revealed by SEM images of the obtained product, as a result of the fast crystal growth (Fig. S5a, b). 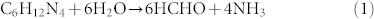

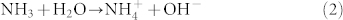


**Figure 3:**
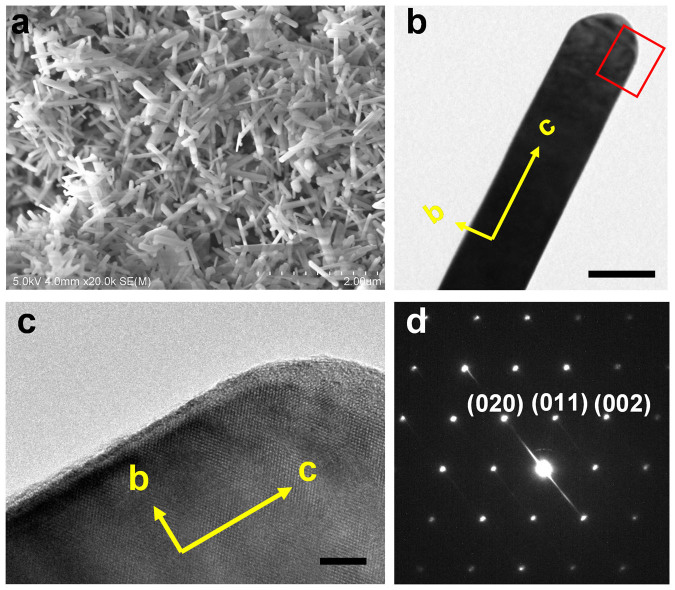
Nanorod particles synthesized with hexamethylenediamine. (a) SEM image (scale bar = 2 μm); (b) TEM image (scale bar = 50 nm); (c) HRTEM image (scale bar = 5 nm) and (d) SAED pattern.

In order to control the shape of the synthesized LiCoPO_4_, the hexamethylenediamine was used as structure-directing agent instead of hexamethylenetetramine. In view of molecular structure, HMD is composed of a long alkyl chain, thus providing not only *in situ* OH^−^ source, but also shape-regulating agent. Furthermore, the melting point of HMD is 50°C, thus, enable to mix with ethanol at any ratio at 60°C. When 4 mmol HMD was used, a similar nanoparticles were synthesized as shown in SEM and TEM images in Fig. S5c, d. Fig. 3 shows electron microscopy images of LiCoPO_4_ synthesized with 10 mmol HMD. The SEM and TEM images reveal that the obtained sample consists of uniform LiCoPO_4_ nanorods with 500–1,000 nm length and 50 nm thickness. The crystal orientation of LiCoPO_4_ nanorods was further characterized by HR-TEM with SAED on an individual particle and the result is shown in Fig. 3c, d. The HRTEM image of a portion of nanorod in Fig. 3b is displayed in Fig. 3c, revealing the highly ordered single crystalline nature of LiCoPO_4_ particles. The HR-TEM image clearly exhibits the interplanar spacing of (011) atomic planes. The SAED of nanorod exhibits well-defined diffraction spots, indexed to orthorhombic *Pnma* space group. The electron beam is along [100] direction. The SAED pattern confirms that the long axis of the nanorod particle is parallel to the *c* direction as indicated by arrow in Fig. 3b and the particle exposes large {010} and {100} facets along its length.

**Figure 4:**
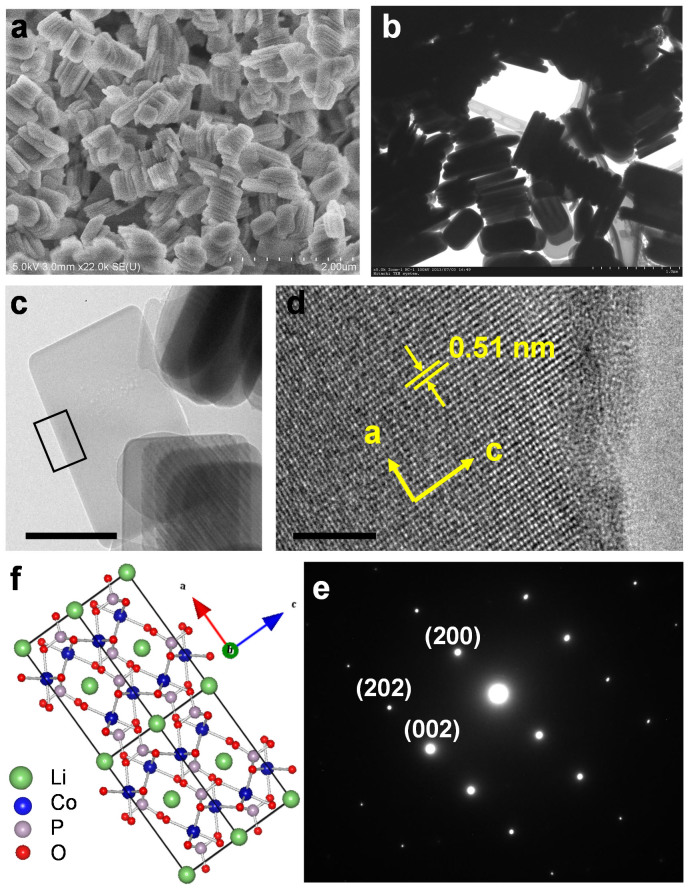
Nanoplate particles synthesized with hexamethylenediamine. (a) SEM image (scale bar = 2 μm); (b) low-magnification TEM image (scale bar = 1 μm); (c) high-magnification TEM image (scale bar = 200 nm); (d) HRTEM image of a portion of the particle shown in (c) (scale bar = 5 nm); (e) SAED pattern and (f) crystal structure of LiCoPO_4_ viewed along [010] direction.

When amount of HMD was increased to 20 mmol, the LiCoPO_4_ nanoplates were obtained which were assembled in superlattices in an oriented manner. As shown in Fig. 4a and b, the product comprises of nanoplates with lengths of 500 nm, widths of 200 nm and a mean thickness of 50 nm. In addition, the TEM image in Fig. 4b suggested that the primary plate-like nanocrystals assembled together in an oriented fashion, producing highly ordered superlattices. The exposed crystal facets of LiCoPO_4_ nanoplates were further studied by HR-TEM and SAED on an individual particle as shown in Fig. 4d and e. The diffraction pattern can be indexed to the [010] zone axis of single-crystal olivine structure, indicating that the incident electron beam is along the *b* direction (Fig. 4e). The electron beam is perpendicular to the lateral plane of the nanoplate, thus, the platelike nanocrystals are exposed {010} facets. The high-resolution TEM image (Fig. 4d) shows the (200) and (020) atomic planes with a lattice spacing of 2.35 Å and 5.1 Å and an interfacial angle of 90°. The observation by TEM is consistent with the corresponding model of atomic arrangement of LiCoPO_4_ olivine structure projected along [010] direction displayed in Fig. 4f. The results show that the thinnest part of the nanoplates is along the *b*-axis, which is favorable direction for the Li ion diffusion. It can also be observed that the ratios between intensities of (010) with (311) diffraction peaks in the XRD pattern of nanoplates are much higher than conventional value (Fig. S1c). The enhancement of (010) peak intensity indicates that the LiCoPO_4_ nanocrystals are dominated by {010} planes. Furthermore, the EDS spectrum of the sample confirms the LiCoPO_4_ phase purity and the EDS elemental mappings by SEM (Fig. S6) reveals the uniform distribution of Co, P, O in the samples.

## Discussion


**Figure 5:**
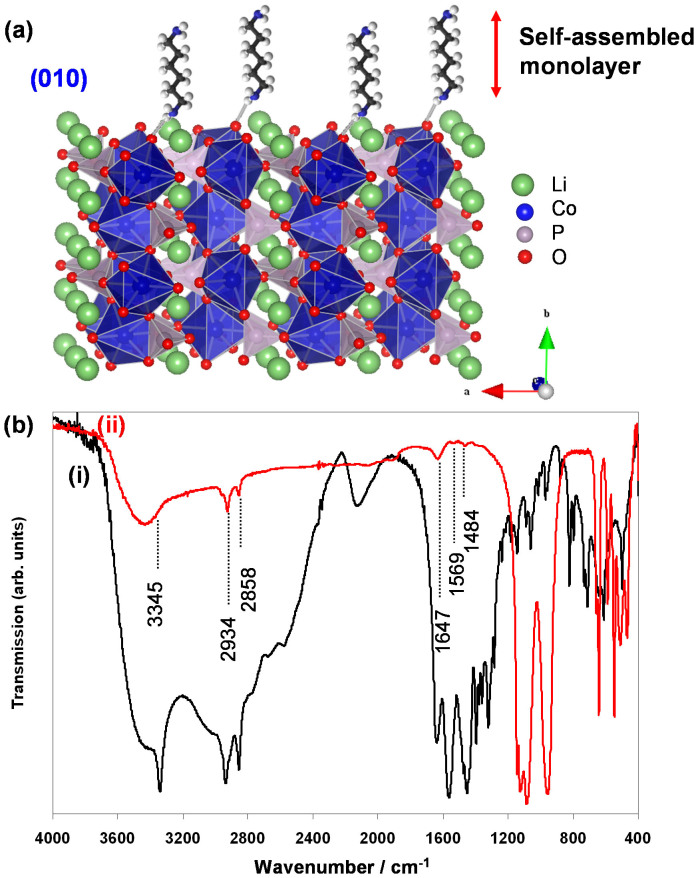
The adsorption of hexamethylenediamine. (a) Schematic illustration of the adsorption configuration of hexamethylenediamine on {010} facets of the LiCoPO_4_ olivine and (b) FTIR spectra of (i) hexamethylenediamine, (ii) nanoplate particles.

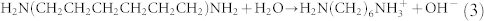
Based on above results, one may understand that HMD played role as a *in situ* OH− agent through their dissociation in the solution similar to the role of HMT discussed above (Eq. 3). Beside that, HMD also acted as shape-controlling agent to regulate the crystallographic orientation of the LiCoPO_4_ nanocrystals. Accordingly, the formation of nanorods and nanoplates can be understood on the basis of capping chemistry and crystal growth habits of LiCoPO_4_. In hydrothermal/solvothermal growth, the adsorption of additive can change the order of the free energies of different facets. The reduction of the interface tension of specific facets due to adsorption of additives would stabilize the facets and facilitate the growth of such facets. HMD might selectively adsorb on {010} facet through the N–H···O hydrogen bonds with oxygen atoms on LiCoPO_4_ surfaces. Therefore, the growth along [010] is suppressed, leading to growth of rodlike particles with favorable *c* direction growth or platelike particles with exposed {010} facets. The selective adsorption of HMD on {010} facets can be understood through the crystal structure of LiCoPO_4_. Co ions on the {100} plane are coordinated by PO_4_^3−^ groups, thus, minimize the surface energy of this facet. In contrast, Co ions with dangling bond are observed on high-energy {010} and {001} surfaces. However, the atomic density on the {010} facets with oxygen-rich boundary is greater than that on the {001} plane (Fig. S7). HMD might therefore prefers to adsorb on the {010} facets which offer more oxygen atoms for N–H···O hydrogen bonds (Fig. 5a). Furthermore, the Co-Co atoms distance on {010} facets (5.6 × 4.7 Å) is shorter than that on {001} facets (10.2 × 5.9 Å), which would be appropriate space for self-assembled monolayer of HMD due to their hydrophobic attraction. As a result, the growth of {010} facets became predominant and the development of the {001} or {100} planes were restrained.

In order to verify the proposed mechanism, Fourier transform infrared spectroscopy (FTIR) spectra of the synthesized LiCoPO_4_ particles were investigated. Fig. 5b shows the FTIR spectra of nanorods and nanoplates (synthesized with HMD) in comparison with that of HMD (Table 1). The FTIR peaks of PO_4_^3−^ can be found such as a symmetric stretching mode at 971 cm^−1^, a doublet at around 468 cm^−1^ and two triplets in the region 1,055–1,106 cm^−1^ and at 641 cm^−1^. More importantly, the peaks around 2,934 cm^−1^ and 2,859 cm^−1^ (observed on spectra of nanorods and nanoplates) can be assigned to the asymmetric and symmetric stretching vibrations of C–H of the alkyl chain. Moreover, the absorption band at 3,445 cm^−1^ was broadened due to the interaction of hydrogen bonds –NH**···**O. The peak at 1,569 cm^−1^ is attributed to the bending vibration of –NH_2_. It is suggested that –NH_2_ coordinates with Co(II) on the surface of the particles. These absorption bands are not observed in the FTIR spectra of particles synthesized with HMT or after calcination (Fig. S8). The FTIR results verified that HMD molecules were adsorbed on the surface of LiCoPO_4_ and may play an important role on the formation of the high aspect ratio rodlike and platelike nanostructures and their assembly.

The self-assembly of nanoplates into superlattices can be understood from the unique structures of hexamethylenediamine. As discussed above, HMD may bind to oxygen atom on the surface of the LiCoPO_4_ nanocrystals *via* hydrogen bonding. Thus, HMD may cover the surface of the primary nanocrystals at {010} facets during the crystal growth prohibiting further growth into larger crystals. It should be noted that HMD has two amine groups located at molecular ending. Therefore, HMD may provide hydrogen bonding for interconnected LiCoPO_4_ nanocrystals, with both ends bound to the surface of the primary nanocrystals (Fig. 1). Although, it is difficult to confirm this binding configuration by the FTIR spectra. The further structural analysis may confirm this proposed binding model. The similar oriented assembly of nanocrystals into superlattices has also been observed in supercritical hydrothermal synthesis^41^. It is also possible that the oriented arrangement of the primary nanocrystals occurs to minimize the interfacial strain energy. The assembly of such well-developed facet particles is energetically and entropically favoured because the maximize face-to-face contact may reduce the surface tension^42^. Many ligand molecules have been found to enable the stabilization of nanocrystals in solution and provide mutual interaction via hydrocarbon chains to generate an effective attraction between the nanocrystals^41^,^42^,^43^.

The amount of HMD on the surface of LiCoPO_4_ nanocrystals can be estimated from TGA (Fig. S9). TGA curves show a weight loss from 300 to 700°C with exothermic peak, which is attributed to the combustion of organic molecules. This results further confirm that LiCoPO_4_-HMD are bound through chemical bonds rather than *via* physisorption.

### Electrochemical performance of LiCoPO_4_ nanocrystals

To prepare the carbon coating (about 3 wt.%), the LiCoPO_4_ particles were mixed with 10 wt.% sucrose in ethanol, then the dry mixture was carbonized at 650°C for 1 h in Ar atmosphere. It should be noted that higher amount of sucrose resulted in the reduction of lithium metal phosphate to form cobalt phosphide due to the carbothermal reduction during heat-treatment^44^ (Fig. S10). The SEM images of the LiCoPO_4_/C particles reveal that the morphology of particles was retained after the heat treatment (Fig. S11). In addition, Raman spectra of the coated samples showed D band at 1,354 cm^−1^ and G band at 1,570 cm^−1^, revealing the presence of an amorphous carbon layer covered the surface of the LiCoPO_4_ particles (Fig. S12). The electrochemical performance of the synthesized LiCoPO_4_/C has been measured and investigated by galvanostatic charge-discharge method. The recent studies showed that the hydro/solvothermal or low-temperature synthesized olivine LiMPO_4_ suffers from the cation exchange antisite defects^45^,^46^,^47^. The occupation of Li sites by Co cations unexpectedly block the lithium transport along the *b* direction, resulting in the fading of the capacity^45^,^46^,^47^. It was suggested that a post-heat treatment temperature can be used to eliminate cation disorder^48^. Therefore, the carbonization step in inert atmosphere at 650°C for 1 h may also improve the ordering of cations in the frameworks. Particularly, the sample after heating at high temperature show improvement in both discharge voltage and discharge capacity (Fig. S13).

**Figure 6:**
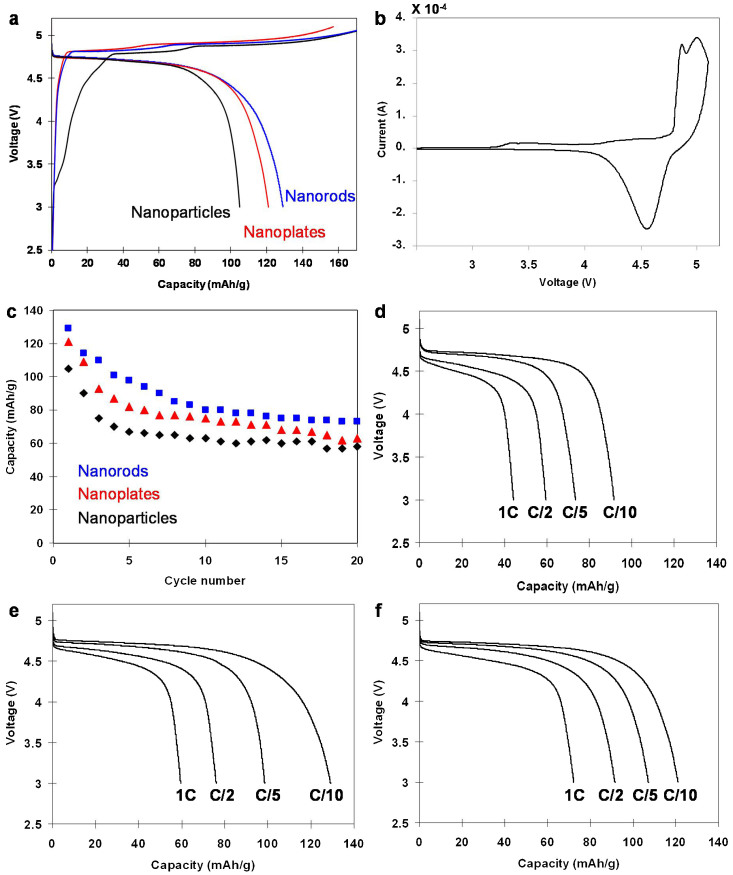
Electrochemical performances of the synthesized LiCoPO_4_ in Li-ion batteries tested in the potential range of 3.0–5.1 V. (a) typical first charge/discharge profiles; (b) cyclic voltammograms of the cells containing nanorod LiCoPO_4_; (c) cyclic performance of LiCoPO_4_ at 0.1 C rate. The initial discharge curves of LiCoPO_4_ at different current rates: (d) nanoparticles; (e) nanorods and (f) nanoplates.

The typical charge and discharge curves of the LiCoPO_4_/C samples at 0.1 C rate with cut-off voltage window of 3.0 to 5.1 V are shown in Fig. 6a. All the samples possess two voltage plateaus, indicating two-step lithium deintercalation. The cyclic voltammograms of the cell containing LiCoPO_4_ shows two oxidation peaks at 4.8 V and 4.9 V versus Li/Li^+^ and one reduction peak at 4.60 V (Fig. 6b), in agreement with cyclic voltammograms reported by other groups^49^,^50^,^51^. This two-step behavior of lithium extraction from LiCoPO_4_ was associated with the transitions: LiCoPO_4_ ↔ Li_0.7_CoPO_4_ and Li_0.7_CoPO_4_ ↔ CoPO_4_^16^,^51^.

All the LiCoPO_4_ samples exhibited a wide and flat voltage plateau at around 4.75 V versus Li/Li^+^ with a initial discharge capacity up to 130 mAhg^−1^ at 0.1 C rate for LiCoPO_4_ nanorods. The discharge capacity of the nanoparticles and nanoplates is 105 mAhg^−1^ and 121 mAhg^−1^, respectively. The discharge capacity of nanoplates and nanorods are higher than that of nanoparticles. The higher discharge capacity of the nanoplates and nanorods is presumably due to their crystallographic orientation. The nanorods with *b* axis along the shortest dimension or nanoplates exposed {010} facets result in short lithium ion diffusion lengths and enhance the diffusion rate. The nature of nanorods also promoted fast charge transport^52^. Nevertheless, the discharge capacity is only 77.8% of the theoretical capacity. The low discharge capacity of the obtained material is believed due to the presence of antisite defects^47^. The partly occupation of Li sites by Co atoms inevitably blocks the lithium ion diffusion pathway, thus, only partly Li ions were extracted and reinserted into the frameworks.

The cyclic performance of the LiCoPO_4_/C samples were also evaluated and the result is shown in Fig. 6c and Fig. S14. The discharge capacity of LiCoPO_4_ nanorods is 73 mAhg^−1^ at 20^th^ cycle with a current rate of 0.1 C and the capacity retention is 56.6% of initial discharge capacity. The great capacity fading were observed in the first 5^th^ cycles, and then the discharge retentions gradually become stable. The LiCoPO_4_ cathodes have generally suffered from poor cycling stability^51^. It was also demonstrated that the capacity fading of LiCoPO_4_ is due to the instability of electrolyte at 5 V. The side reactions between electrolyte and electrode may produce a solid-electrolyte interface (SEI)^53^, leading the lithium loss. Recently, Dimesso et al. performed the ac-impedance measurement of LiCoPO_4_ particles after cycling to reveal the formation of SEI-layers which hinder the kinetics of the (de)intercalation processes^53^. Fig. 6d–f present the rate capacities of the cells containing LiCoPO_4_ at various discharge rates ranging from 0.1 C to 1 C. At high discharge rate of 0.5–1 C, the cell exhibited a reduced discharge voltage at 4.5–4.4 V. Notably, the nanoplates delivered highest capacity at high rate, although nanorod particles showed better discharge capacity at 0.1 C rate. The nanorods exhibited initial discharge capacity of 98, 76, 60 mAhg^−1^ at 0.2, 0.5, 1 C, respectively, while nanoplates delivered the capacity of 107, 92, 72 mAhg^−1^ at 0.2, 0.5, 1 C, respectively. The result is in good agreement with previous report that the nanoplates with exposed {010} facets showed the enhanced rate capacity^54^,^55^. Furthermore, the oriented aggregation of nanoplates in superlattices facilitated the charge and mass transfer between particles and increase the tap density of overall particles^56^,^57^,^58^. The result indicates the important impact of crystal orientation on the rate capacity of the cathode materials.

In summary, we demonstrated an one-pot supercritical fluid processing for the controllable synthesis of LiCoPO_4_ nanoparticles, nanorods and nanoplates. The amines played important roles as *in situ* OH^−^ source and shape-directing agent for tuning the crystal orientation. Nanorods and nanoplates with dominant {010} surfaces have been obtained with the aid of hexamethylenediamine. Inspired by the unique shape with short transport path length, the LiCoPO_4_ particles were used as cathode materials for lithium batteries, and the cells exhibited initial discharge capacities up to 130 mAhg^−1^ at 0.1 C rate. The nanoplates have superior rate capacity owing to their unique structural features and assembly. We believe that the LiCoPO_4_ material with an optimal size and appropriate crystal orientation may have good potential for LIBs with high energy density and high power density.

## Methods

### The synthesis of LiCoPO_4_ materials

LiCoPO_4_ materials were directly synthesized by one-pot supercritical fluid method with a reaction time of 6 min. Typically, 2 mmol cobalt acetate tetrahydrate (Co(Ac)_2_.4H_2_O, Wako, Japan) and 2 mmol lithium acetylacetonate (LiAcac, Wako, Japan) was dissolved in 15 ml ethanol and the solution was heated at 60°C with continuous stirring. The solid salts was completely dissolved within 1 min, followed by addition of solution of phosphoric acid (H_3_PO_4_, aqueous solution, 85%, Wako, Japan) in 4 ml ethanol. The molar ratio of Co(Ac)_2_:LiAcac:H_3_PO_4_ is of 1:1:1. Hexamethylenetetraamine (HMT) or hexamethylenediamine (HMD) were used as *in situ* OH^−^ sources and structure-directing agent to control the growth rate as well as morphology of LiCoPO_4_. The obtained blue suspension was heated with continuous stirring at 60°C for 10 munities then 5 ml each solution was transfer to a batch reactor (10 ml, 4 reactor) and heated at 400°C and 38 MPa pressure for 6 minutes. After which reactors were allowed to cool to room temperature by water quenching. The resultant powder was separated by centrifugation and washed with distilled water and ethanol until the pH of the solution became neutral. Finally, the obtained specimen was dried at 60°C for 1 day. The above synthesis condition has been optimized to obtain the LiCoPO_4_ particles with good homogeneity and superior cathode performance.

### Materials characterization

The crystalline phase of the samples were characterized using powder X-ray diffraction (Rigaku RINV-2200, 40 kV and 30 mA) with CuK*α* radiation (*λ* = 1.5406 Å). Data were collected in the 2θ-θ scanning mode with a scan speed of 4° min^−1^ and a step size of 0.02°. The morphology of particles was observed using field-emission SEM (Hitachi S-4800 with EDS) at an accelerating voltage of 5 kV. TEM (Hitachi H7650) and high-resolution TEM (JEOL JEM 2100F, 200 kV and TOPCOM EM-002B, 200 kV) were conducted using specimens dispersed in ethanol and then dropped onto Cu microgrid coated with a holey carbon film, followed by the evaporation of the ethanol. Elemental mapping, energy dispersive spectroscopy were observed using STEM JEOL JEM-2100F. IR spectra were recorded with a FT/IR 6200 spectrophotometer (JASSO, Japan) within the range of 400–4000 cm^−1^. Samples in the solid state were measured in KBr matrix pellets were obtained with hydraulic press under 40 kN pressure. Raman spectra were evaluated in the range of 400–2000 cm^−1^ using NRS-3100, JASSO, Japan.

### Electrochemical measurements

The electrochemical performance of LiCoPO_4_ was investigated using coin-type cells (CR2032, Fig. S15). The working electrodes is composed of 80 wt.% LiCoPO_4_, 10 wt.% PTFE (poly(tetrafluoroethylene)) as a binder and 10 wt.% acetylene black. These materials were ground by conventional agar motor to make electrode paste. The prepared paste was spread uniformly, rolling into sheet then dried in a vacuum oven for 4 h at 160°C. The cathode sheet was punched into circular discs and cut into wafers (7 mm in diameter, 0.025 mm in thickness, 5–6 mg). The tested cell was assembled inside an argon-filled glove box. For electrochemical measurements, the cell is composed of lithium metal counter, reference electrodes and a LiCoPO_4_ positive electrode. The cathode and reference electrodes were separated by a microporous polypropylene film. 1 M solution of LiPF_6_ in a mixed solvent of ethylene carbonate (EC) and dimethyl carbonate (DMC) with 1:1 in volume ratio (Tomiyama Pure Chemical Co., Ltd.) was used as the electrolyte. The charge-discharge cycling were performed galvanostatically between 3.0 and 5.1 V versus Li/Li^+^ on multi-channel battery testers (Hokuto Denko, Japan) at various charge/discharge rates ranging from 0.1 to 1 C (1 C = 167 mAhg^−1^). Current densities and specific capacities were calculated on the basis of the weight of LiCoPO_4_ cathode in the electrode.

## Author Contributions

Q.D.T. and I.H. conceived and designed this work. Q.D.T. and M.K.D. carried out the synthetic experiments and conducted the electrochemical test. Q.D.T., Y.G. and T.T. performed HRTEM, TGA, Raman and FTIR measurements. Q.D.T. wrote the paper; all the authors participated in analysis and discussion of the results.

## Supplementary Material

Supplementary Information Controlling The Shape of LiCoPO_4_ Nanocrystals by Supercritical Fluid Process for Enhanced Energy Storage Propertie

## Figures and Tables

**Table 1:** Infrared vibrational assignments

Vibrational modes		
LiCoPO_4_	hexamethylenediamine	Frequency (cm^−1^)
	*ν*_as_(NH_2_), *ν*_s_(NH_2_)	3,390; 3,345
	*ν*_as_(C–H), *ν*_s_(C–H)	2,934; 2,859
	*δ*(NH_2_)	1,569; 798
	*δ*(CH_2_)	1,484
triplet *ν*_as_(PO_4_)		1,085–1,144
	*δ*(C–N)	1,071
singlet *ν*_s_(PO_4_)		971
	*δ*(C–C)	722
triplet *δ*_as_(PO_4_)		641
doublet *δ*_s_(PO_4_)		468

*ν*_as_ = asymmetric stretching vibration; *ν*_s_ = symmetric stretching vibration; *δ* = bending vibration.
