# Supplementary material for: Controlling the shape of LiCoPO4 nanocrystals by supercritical fluid process for enhanced energy storage properties
Source: Sci Rep. 2014 Feb 5;4:3975. doi: 10.1038/srep03975 (PMC3913972; doi:10.1038/srep03975)
Supplement: Supplementary Information — Controlling The Shape of LiCoPO4 Nanocrystals by Supercritical Fluid Process for Enhanced Energy Storage Propertie [file srep03975-s1.pdf]

Supplementary Information

Controlling the Shape of $LiCoPO_4$ Nanocrystals by Supercritical Fluid Process for Enhanced Energy Storage Properties

Quang Duc Truong*, Murukanahally Kempaiah Devaraju, Yoshiyuki Ganbe, Takaaki Tomai, Itaru Honma*

Institute of Multidisciplinary Research for Advanced Materials, Tohoku University, Sendai 980-8577, Japan.

**Email address*: tqduc@mail.tagen.tohoku.ac.jp; i.honma@tagen.tohoku.ac.jp.

Tel./Fax: 81-22-217 5816.

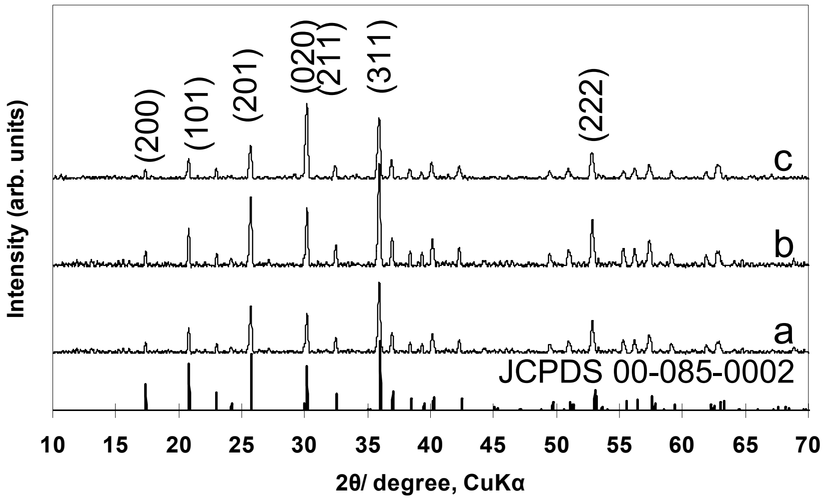

Supplementary Figure S1. XRD patterns of the synthesized $LiCoPO_4$ nanocrystals by supercritical processing using (**a**) 4 mmol hexamethylenetetramine and (**b**, **c**) 10 mmol and 20 mmol hexamethylenediamine, respectively.

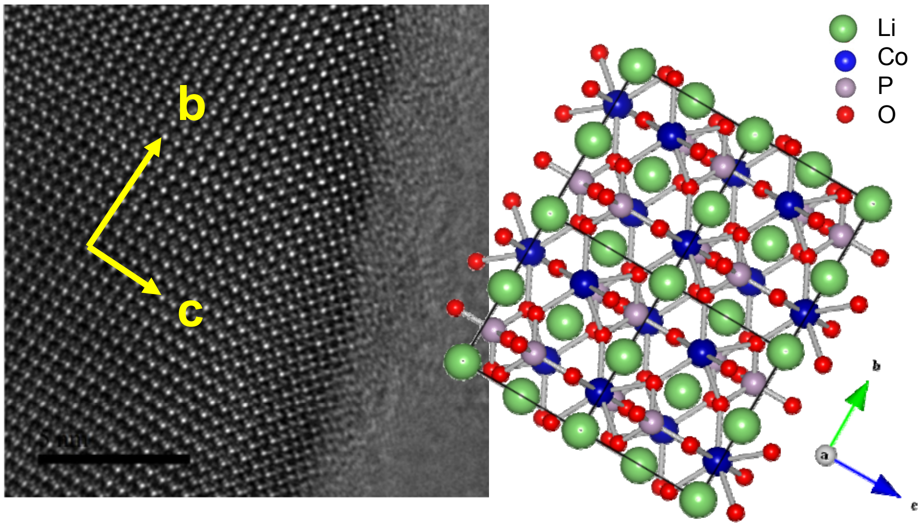

Supplementary Figure S2. TEM image of nanoparticle (scale bar = 5 nm) and the inset shows the schematic illustration for the $LiCoPO_4$ olivine structure view along [100] direction.

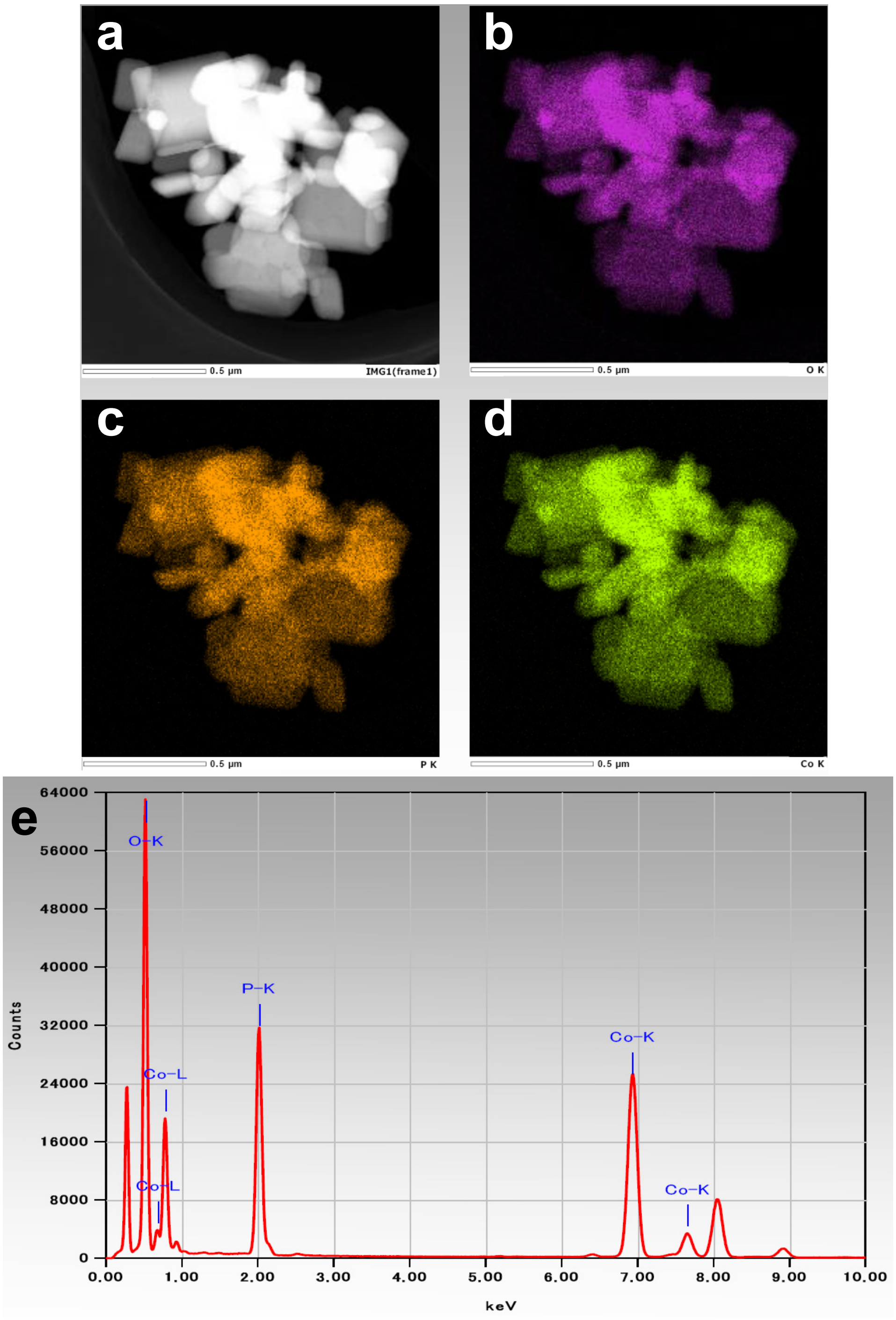

Supplementary Figure S3. (**a**) STEM image; (**b**, **c**, **d**) elemental mapping of O, P, Co, respectively and (**e**) EDS spectrum of the synthesized $LiCoPO_4$ nanoparticles by STEM analysis.

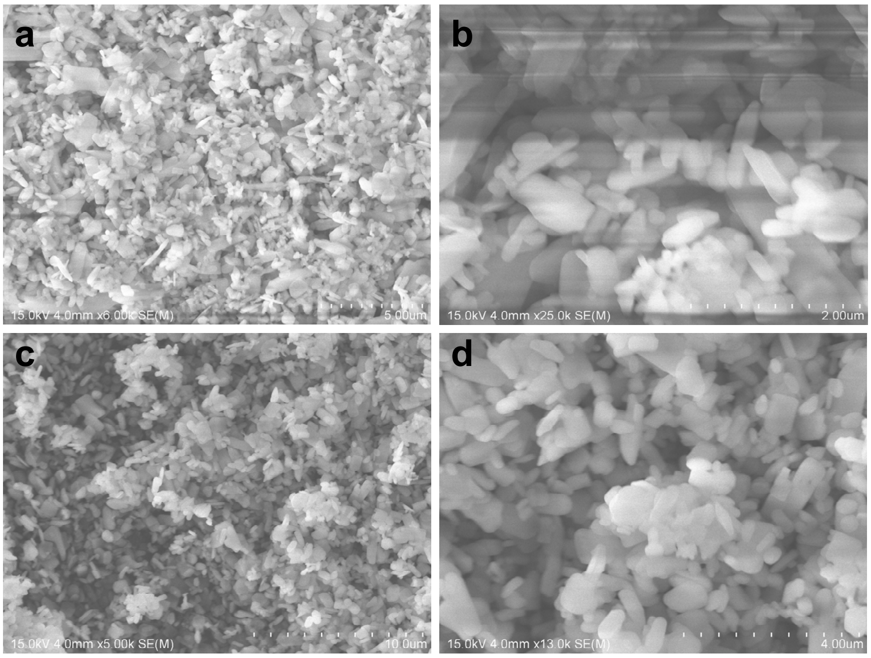

Supplementary Figure S4. SEM images of (**a**, **b**) particles synthesized with 2 mmol of HMT and (**c**, **d**) particles synthesized with 10 mmol of HMT.

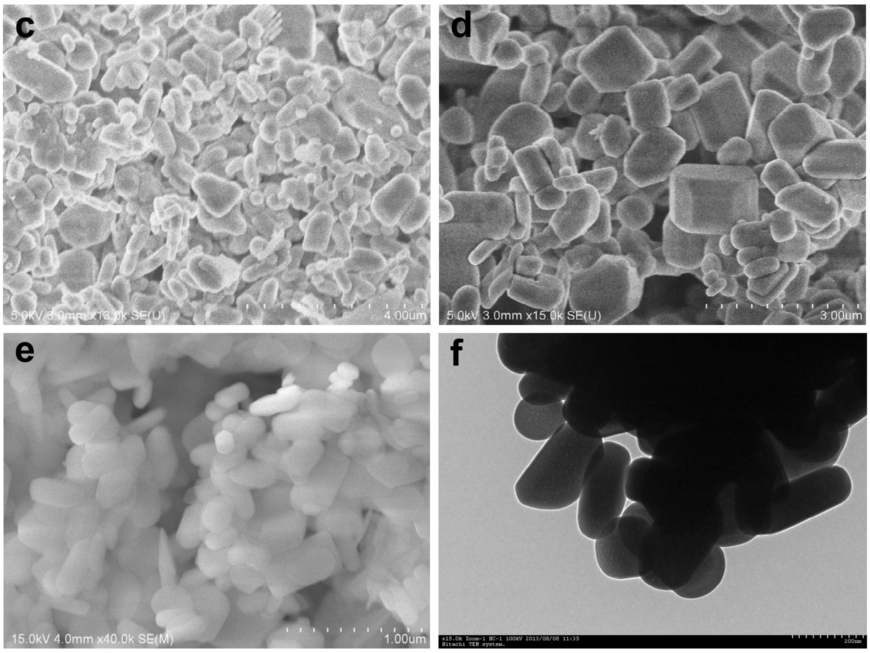

Supplementary Figure S5. SEM images of (**a**, **b**) particles synthesized with 0.5 mL NH_3 and 1 mL NH_3 solutions respectively and (**c**, **d**) SEM and TEM images of particles synthesized with 4 mmol hexamethylenediamine.

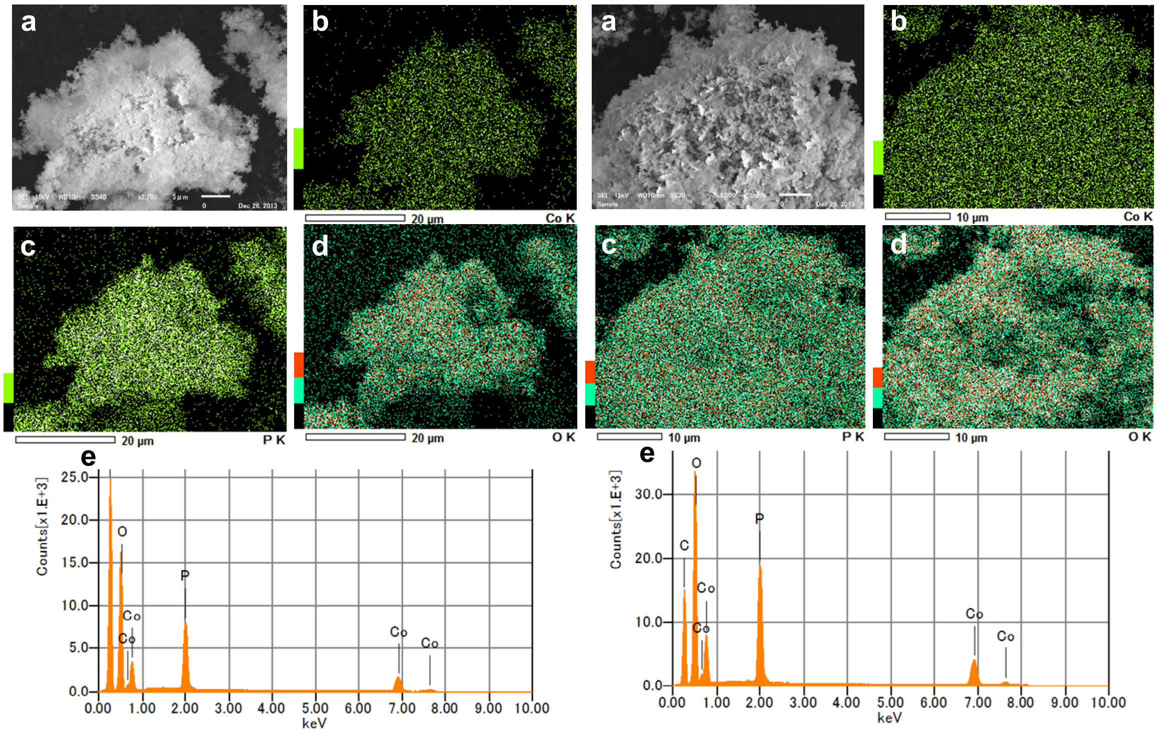

Supplementary Figure S6. (**a**) SEM image; (**b**, **c**, **d**) elemental mapping of Co, P, O respectively and (**e**) EDS spectrum of the synthesized $LiCoPO_4$ nanorods (left); nanoplates (right) by SEM analysis.

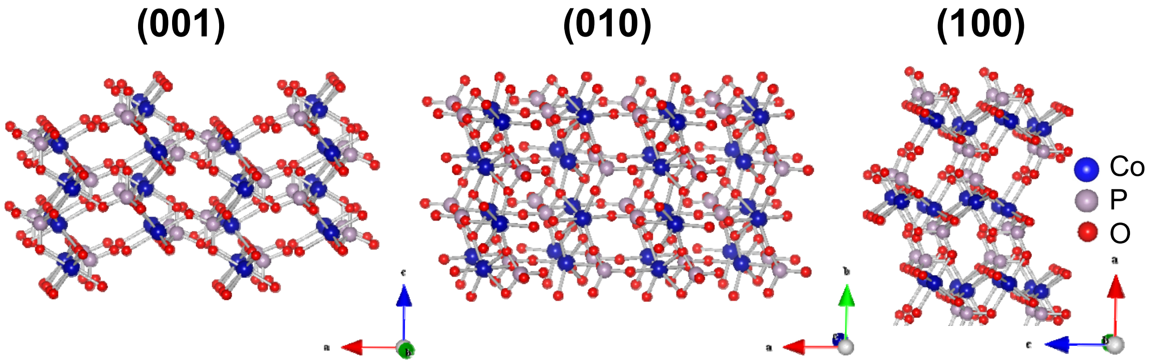

Supplementary Figure S7. Schematic illustrations for the $LiCoPO_4$ olivine structure viewed along different directions. Li atoms are omitted for clarify.

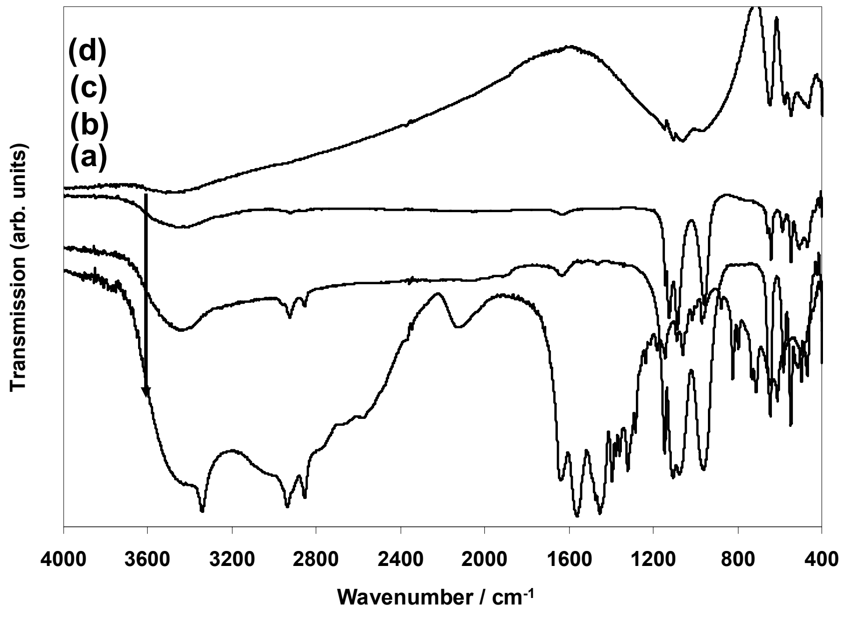

Supplementary Figure S8. FTIR spectra of (**a**) hexamethylenediamine; (**b**) nanorod particles; (**c**) nanoparticles synthesized with HMT and (**d**) nanoplate particles after calcination (bare $LiCoPO_4$).

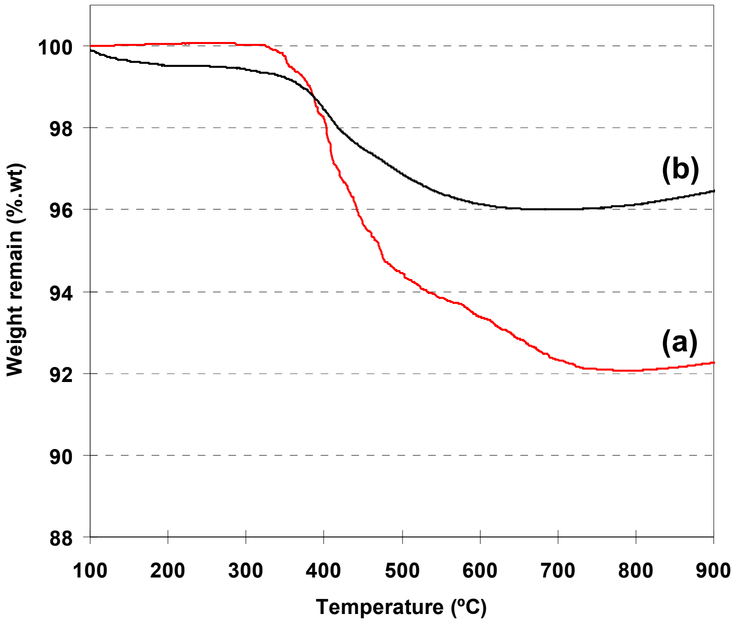

Supplementary Figure S9. TGA curves of (**a**) nanoplate particles and (**b**) nanorod particles.

The amount of hexamethylenediamine in the products ranges from 3.4 wt % in nanorods to 7.9 wt % in nanoplates. Considering the shape of the particles observed by microscopy images and the specific surface area (BET), we have calculated the number of amine molecules attached to the surface and the surface coverage (Table S1). The percentage of {010} surfaces were calculated to be 48 % for nanorods (500–1000 nm length and 50 nm thickness) and 74 % for nanoplates (with lengths of 500 nm, widths of 200 nm and a mean thickness of 50 nm). To estimate the surface coverage, lattice constant of olivine structure of $LiCoPO_4$ on the {010} facets was used as a = 10.2045 Å, c = 4.7002 Å. Then, percentage of surface coverage was calculated as the ratio of the number of amine molecules to the number of cobalt atoms on the {010} surface.

Table S1. Surface coverage of $LiCoPO_4$ nanocrystals

	BET (m^2 g^{-1})	Weigh loss (%)	Molecule (nm^{-2})
Nanorods	22.4	3.4	1.64
Nanoplates	16.7	7.9	3.31

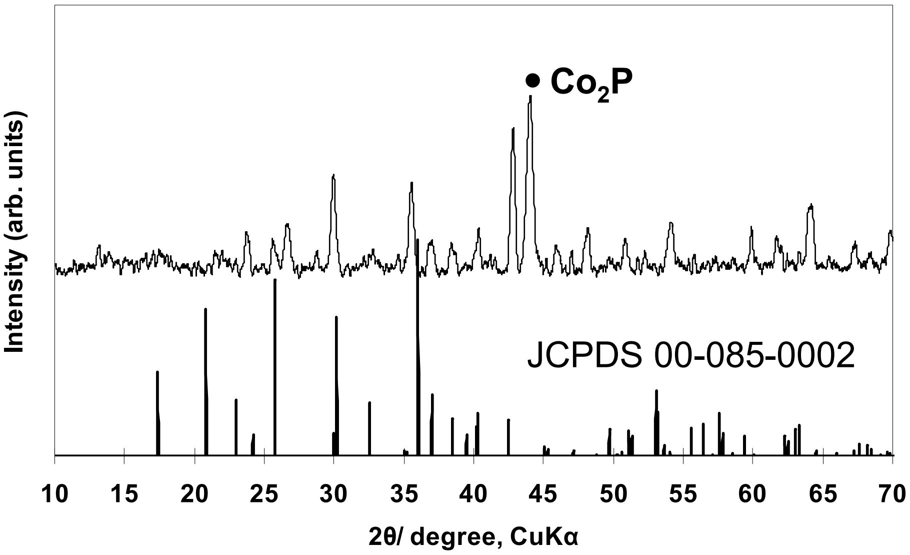

Supplementary Figure S10. XRD pattern of particles obtained after the carbothermal reduction with large amount of sucrose (> 20 wt.%).

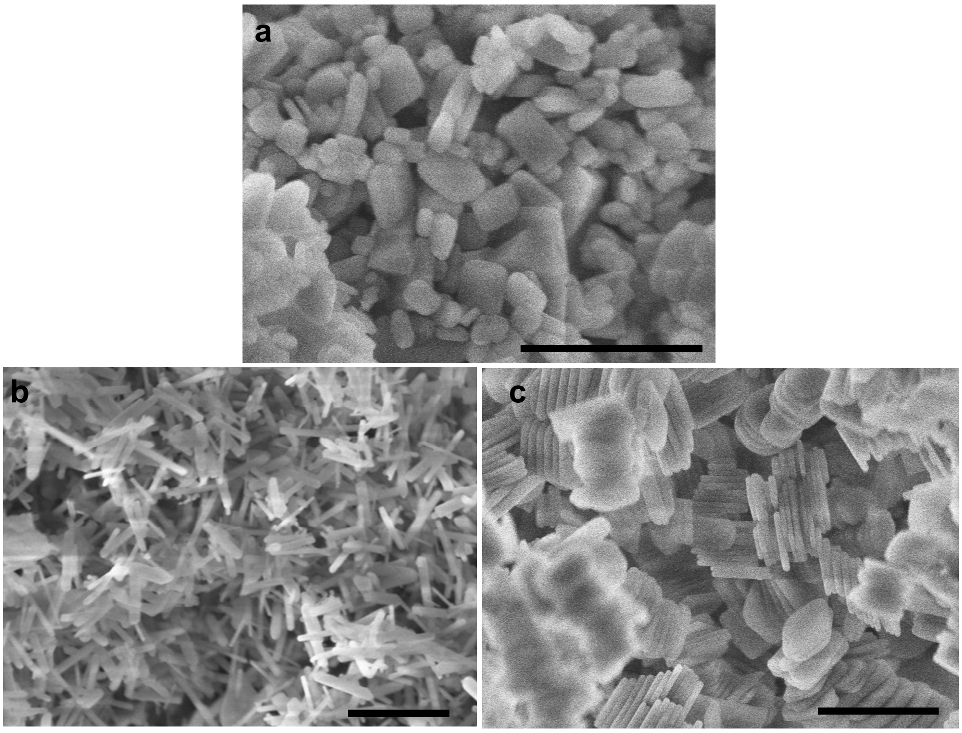

Supplementary Figure S11. SEM images of particles after heat treatment with carbon coating: (**a**) nanoparticles; (**b**) nanorods and (**c**) nanoplates (scale bar = 1 μm).

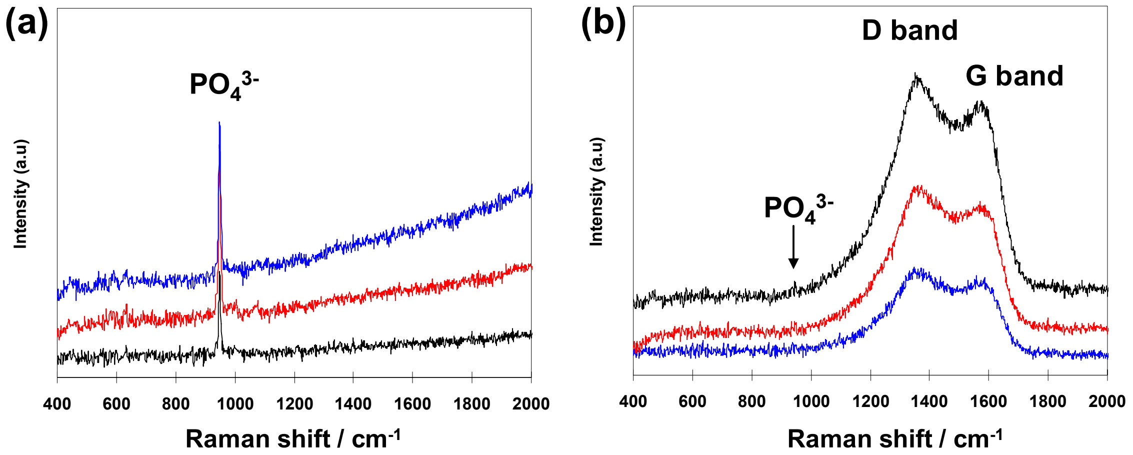

Supplementary Figure S12. Raman spectra of (**a**) the as-synthesized particles and (**b**) $LiCoPO_4$/C. Dark curves: nanoparticles; red curves: nanorods; blue curves: nanoplates.

The major features, commonly observed in carbon materials, are the D band at 1354 cm^{-1} and G band at 1570 cm^{-1}. The G band at 1570 cm^{-1} corresponds to an E*2g* mode related to the sp2-bonded carbon atom's vibration. And the D band at 1354 cm^{-1} arises from a breathing mode of κ-point phonons of A_{1g} symmetry. The bands located at 948 cm^{-1} can be assigned to intramolecular stretching modes of PO_4^{3-}.

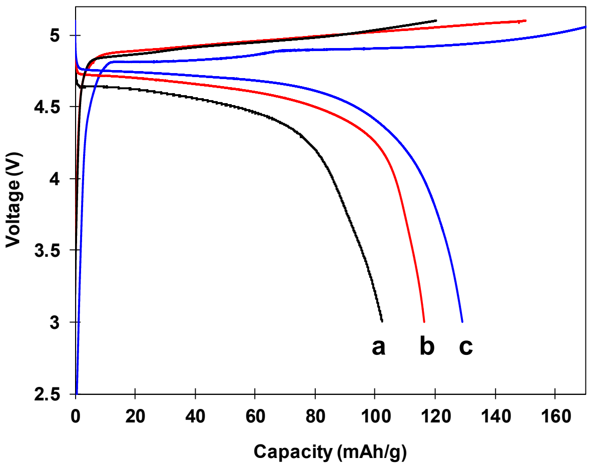

Supplementary Figure S13. The first charge/discharge curves of cell containing nanorods: (**a**) as-synthesized particles; (**b**) heat treatment at 450 °C and (**c**) heat treatment at 650 °C.

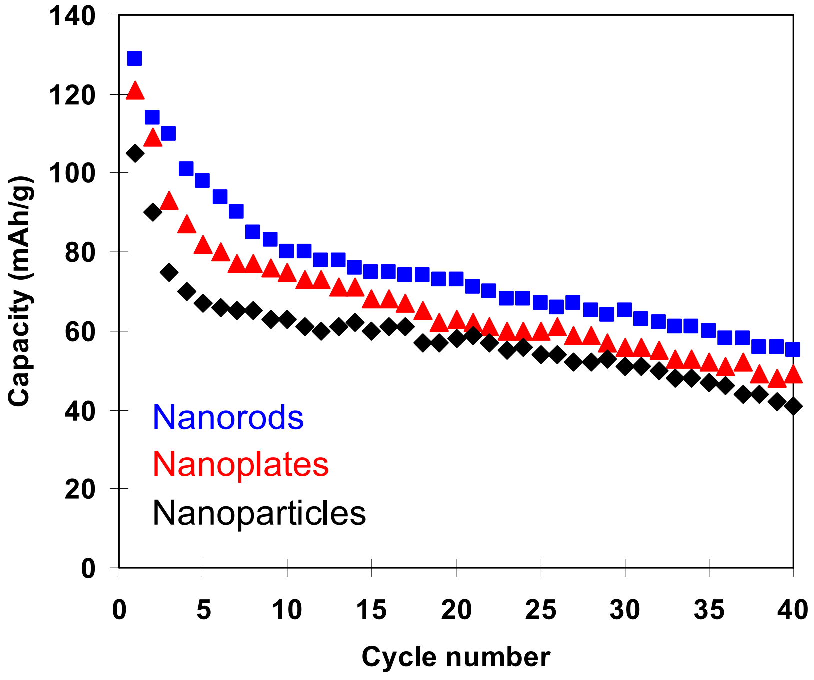

Supplementary Figure S14. Cyclic performance of $LiCoPO_4$ at 0.1 C rate up to 40 cycles.

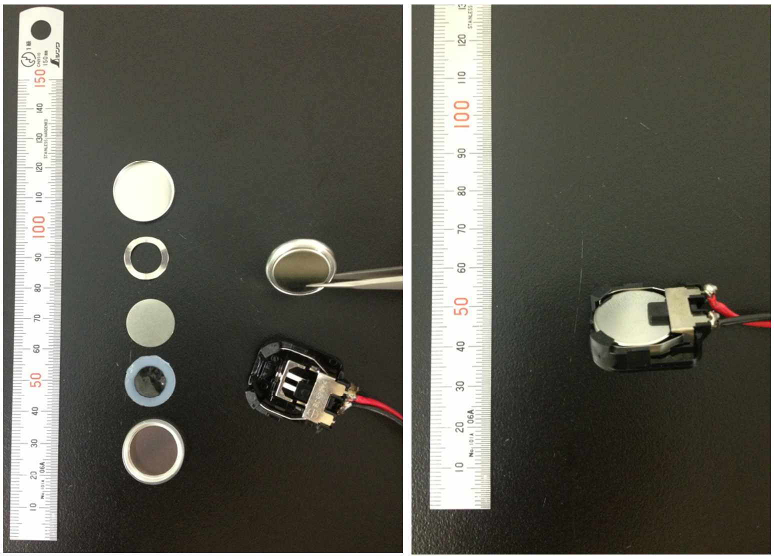

Supplementary Figure S15. Photograph images of the coin cell before and after assembly.
